# „Selten Gehörte“ für partizipative Gesundheitsforschung gewinnen: Herausforderungen und Strategien

**DOI:** 10.1007/s00103-020-03269-7

**Published:** 2020-12-28

**Authors:** Ina Schaefer, Susanne Kümpers, Tina Cook

**Affiliations:** 1 Alice Salomon Hochschule Berlin, Alice-Salomon-Platz 5, 12627 Berlin, Deutschland; 2 Fachbereich Pflege und Gesundheit, Hochschule Fulda, 36037 Fulda, Deutschland; 3 Faculty of Liberal Arts, Education and Social Science, Liverpool Hope University, Liverpool, Großbritannien

**Keywords:** Partizipative Gesundheitsforschung, Selten Gehörte, Inklusion, Partizipation, Zugang, Participatory health research, Seldom heard, Inclusion, Participation, Recruitment

## Abstract

**Hintergrund:**

Die partizipative Gesundheitsforschung gewinnt im Bereich Public Health zunehmend Anerkennung. Dabei werden Menschen, deren Leben oder Arbeit im Mittelpunkt der jeweiligen Forschung stehen, in die Projekte eingebunden. Die Integration des Wissens und der Perspektiven von Menschen, die bisher nur selten gehört wurden, eröffnet dabei die Möglichkeit, gesundheitliche Chancengleichheit in den jeweiligen Lebenswelten zu stärken. Partizipative Gesundheitsforschung (PGF) wird häufig mit Gemeinschaften (Communitys) durchgeführt. Jedoch stellt es eine besondere Herausforderung dar, Personen zu erreichen, die kaum oder keinen Zugang zu Netzwerken oder Angeboten haben. Deren Lebensbedingungen und -qualität müssen in der Forschung berücksichtigt werden. Im vorliegenden narrativen Übersichtsartikel wird diese Problematik beleuchtet und Lösungsansätze für die Forschungspraxis werden entwickelt.

**Methodik:**

Es wurde auf eine umfangreiche Literaturrecherche der Katholischen Hochschule für Sozialwesen Berlin nach deutschsprachigen Artikeln zu partizipativer Forschung zurückgegriffen; ergänzend wurde internationale Literatur gesichtet. Darüber hinaus wurden Prozesse von 3 partizipativen Forschungsprojekten ausgewertet.

**Ergebnisse und Diskussion:**

Die dargestellten Zugangswege zu „selten Gehörten“ beziehen sich auf die Reduzierung systemischer Barrieren und die Verbesserung lokaler Voraussetzungen. Beispielsweise wird vor Beginn der Forschung im engeren Sinn eine Vorbereitungsphase empfohlen. Diese kann für den Beziehungs- und Kooperationsaufbau genutzt werden, um die Distanz der Menschen aus der Lebenswelt gegenüber Forschung zu überbrücken.

## Abstract (en)

**Background:**

Participatory health research is gaining increasing recognition in the public health sector. The people whose lives or work are central to wicked issues around health inequalities are integrated into the projects. Including diverse forms of knowledge and perspectives has the capability of strengthening health equity in specific settings. That proffers agency to the voices of those who are seldom heard. Often, participatory health research (PHR) is done with communities. Finding the truly isolated – those who are not connected to others or to services, the seldom heard – presents a challenge for research. Without hearing the voices of those most distanced from mainstream discourse we run the risk of misunderstanding issues pertinent to their lives. This narrative overview highlights this problem and aims to contribute a solution approach for research practice.

**Methods:**

An extensive literature review for participatory research references in the German language was used and the international literature was also screened. Furthermore, the processes of three participatory research projects were evaluated.

**Results/Discussion:**

The specified strategies to involve the seldom heard refer to lowered systemic barriers and improved local capacity for their participation. For example, before research can start, time should be given for a preparatory phase. This can be used to invest in relationships and co-operation to bridge the distance to research.

## Einführung

Gesundheitliche Nachteile von Menschen v. a. mit niedrigem sozioökonomischen Status (SES), zu denen diejenigen gerechnet werden, die hier als *selten Gehörte* bezeichnet werden, sind vielfach belegt [1, 2]. Von der epidemiologischen bis hin zur sozialwissenschaftlichen Gesundheitsforschung stellt sich die Herausforderung der geringeren Beteiligung dieser Bevölkerungsgruppen [3–5]. Diese zeigt sich ebenso in der Praxis der Gesundheitsversorgung [6] und der Prävention und Gesundheitsförderung [7]; Bauer spricht von einem „Präventionsdilemma“ [8]. Partizipative Gesundheitsforschung (PGF) nimmt für sich in Anspruch, zur Bewältigung dieser Herausforderung beizutragen [9–13].

International ist belegt, dass sozioökonomische Ungleichheit Auswirkungen auf soziale und gesellschaftliche Teilhabe hat: „… die am meisten zu gewinnen hätten, sind von Entscheidungen im Gesundheitswesen am stärksten ausgeschlossen“ [14]. Auch innerhalb marginalisierter Gruppen zeigt sich dieses Phänomen, so wurde in einer Studie mit Transgenderpersonen gezeigt, dass diejenigen mit einem höheren Sozialstatus häufiger Vertretungs- oder Sprecherfunktionen einnehmen [15].

Ausgehend von bisherigen Analysen, welche Mechanismen einer Partizipation entgegenstehen, zeigt der vorliegende Beitrag, wie in 3 partizipativen Forschungsprojekten auch *selten Gehörte* für eine Beteiligung an Forschung gewonnen werden konnten.

Der Begriff der *selten Gehörten *wurde bewusst gewählt, um Menschen mit niedrigem SES weniger stigmatisierend zu beschreiben. Häufig werden sie als *schwer erreichbar, benachteiligt, marginalisiert* oder *vulnerabel* bzw. mit Attributen wie *Migrationshintergrund* oder über ihren SES beschrieben. Der Begriff *selten Gehörte* betont die Verantwortung, Wege zu ihrer Einbeziehung zu finden, ohne eine negative Etikettierung vorzunehmen. Implizit ist z. B. *schwer erreichbar *mit diskreditierenden Zuschreibungen wie *uninformiert *oder *desinteressiert* verbunden [16–18].

In der PGF gibt es bislang wenig Literatur über die Kontaktaufnahme mit und Motivierung der *selten Gehörten*. Aus der vorhandenen Literatur lassen sich strukturelle und organisatorische Gründe ableiten, warum es nicht gelingt, alle Menschen gleichermaßen in Forschung einzubeziehen.

In diesem Beitrag bereiten wir zunächst Erkenntnisse über Barrieren und Strategien aus der Literatur auf. Wir stellen dann das Vorgehen für die Einbindung selten Gehörter in 3 partizipativen Forschungsprojekten vor. Im Zuge der Diskussion reflektieren wir die Vorgehensweisen, um *selten Gehörte* zu erreichen. Im Fazit werden weitergehende Empfehlungen abgeleitet.

## Methodisches Vorgehen

Zunächst haben wir eine im Auftrag der Katholischen Hochschule für Sozialwesen Berlin 2018 als *Mapping-Review* durchgeführte Literaturrecherche zur partizipativen Forschung im deutschsprachigen Raum (Datenbanken EBSCO Host Datenbanken, AGRIS und PubPsych) genutzt [19]. Die Titel und Abstracts der insgesamt 509 Artikel (1990–2018) dieser Recherche wurden auf den Fokus dieses Beitrags geprüft. Mit dem Suchbegriff „hard to reach“ wurde u. a. via PubMed sowie im Schneeballsystem auch internationale Literatur recherchiert.

Die Projekterfahrungen wurden zunächst innerhalb der jeweiligen Forschungsteams v. a. auf Basis von Protokollen und Feldnotizen reflektiert. Sie wurden dann thesenartig zusammengefasst und während 2 Kolloquien des Forschungsverbundes PartKommPlus (02/2019 und 08/2019) in projektübergreifenden Arbeitsgruppen sowie im Juni 2019 in einem Workshop der Jahrestagung der International Collaboration Participatory Health Research (ICPHR) im kollegialen Diskurs analysiert. Darauf aufbauend, reflektierten und verglichen die Autorinnen zur Entwicklung dieses Beitrags die relevanten Prozesse der dargestellten partizipativen Projekte. Diese variieren hinsichtlich der Settings (Quartier, Kita), der Adressatengruppen (Eltern, Ältere, pflegende Angehörige) und der Arbeitsprozesse. Die Darstellung der Projekte wurde auf die Fragestellung dieses Beitrags begrenzt, sodass hier auf die Prozesse und Ergebnisse der gemeinsamen Wissensgenerierung nicht umfassend eingegangen werden kann (vgl. dazu [20–22]).

## Barrieren und Strategien für den Zugang zu partizipativen Forschungsprojekten

Für das Verständnis der Zugangsproblematik sind vor allem in der Literatur berichtete fördernde und hemmende Bedingungen von Interesse. Voranstellen möchten wir erkenntnistheoretische Aspekte, die Fricker als „epistemische Ungerechtigkeit“ konzeptualisiert [23]. Das Konzept erklärt, wie ein unbewusster und unbeabsichtigter Ausschluss bestimmter Gruppen durch Haltungen von Forschenden sowie durch die kollektiv zur Verfügung stehenden sprachlichen Ressourcen verursacht werden kann. Fricker unterscheidet zwischen „testimonial injustice“ infolge von selektiven Wahrnehmungen bzw. der Zuschreibung von Unglaubwürdigkeit aufgrund (impliziter) individueller Vorurteile der Forschenden und „hermeneutical injustice“, wenn im diskursiven Kontext die Begriffe fehlen, um adäquat über Erfahrungen berichten zu können [23, 24]. Für die partizipative Forschung können diese Erkenntnisse nützlich sein, um die Hintergründe für die Herausforderungen bei der Einbindung selten Gehörter zu verstehen.

Fördernde und hemmende Bedingungen sowie Strategien für den Zugang zu selten Gehörten werden in der Literatur für verschiedene Gruppen dargestellt (v. a. für Menschen mit Migrations- oder Fluchterfahrungen, mit chronischen Erkrankungen bzw. Beeinträchtigungen, mit niedrigem SES) sowie für verschiedene Settings (v. a. Arbeitsplatz, Schule). Wir fokussieren hier auf solche Aspekte, die für die nachfolgenden Forschungsprojekte besonders relevant sind.

### Systemische Barrieren

Neben kulturellen Aspekten werden in der Literatur vielfach individuelle Faktoren als Zugangsbarrieren zu selten Gehörten identifiziert: u. a. Misstrauen und Befürchtungen bezüglich eines fehlenden Nutzens oder eines Eindringens in die Privatsphäre sowie Angst vor Vertrauensmissbrauch [3, 16, 25–27].

In der partizipativen Forschung kommen weitere spezifische Gründe hinzu: Menschen in der Lebenswelt erwarten von einer Beteiligung an Forschung nicht unbedingt mehr als eine Befragung oder einen Test. Cook und Inglis forschten mit Männern mit Lernbehinderung, die sich (zunächst) unter Forschung einen medizinischen Test vorstellten: „Wenn sie Ihnen Blut abnehmen, um herauszufinden, warum Sie krank sind“ [28]. Sie konnten sich nicht vorstellen, dass Forschung „nur Reden sein kann“. Solche Vorstellungen sind insbesondere dort zu erwarten, wo es wenig Erfahrung mit partizipativer Forschung gibt.

Im besten Fall werden Forschungsfragen nicht vorab festgelegt, sodass sich die Forschenden aus der Lebenswelt an deren Definition beteiligen können. Diese Offenheit kann Beteiligung aber auch erschweren, weil die Angesprochenen nicht wissen, worum genau es gehen soll und was von ihnen erwartet wird [14]. Auch kann die Idee der Mitbestimmung aufgrund *verinnerlichter Unterdrückung* als Überforderung erlebt werden [29]. Der partizipative Ansatz setzt voraus, „dass die Menschen aus der Zielgruppe das Wissen, Selbstvertrauen und Durchsetzungsvermögen haben, ihre Interessen zu formulieren und zu vertreten“ [18] oder dies erlernen [30]. Für diejenigen, die an mittelschichtstypische Arbeitsroutinen (z. B. längerfristige Planung und Strukturierung) kaum gewöhnt sind, können diese ausschließend wirken.

Auch administrative Regularien bzw. Verwaltungssysteme können die Bereitschaft zur Teilhabe einschränken. Eine Vergütung der Forschungsleistungen der selten Gehörten kann ggf. zu Abzügen bei Sozialhilfebezug führen. Auch kann das Ausfüllen von Formularen eine Hürde darstellen [27].

### Voraussetzungen und Strategien

Der Aufbau vertrauensvoller Forschungspartnerschaften mit *selten Gehörten* erfordert ein schrittweises Vorgehen sowie Respekt, Wertschätzung und die Bereitschaft zuzuhören; ein sicherer Raum muss entwickelt werden [16, 31–34]. Cook et al. berichten über Vorbereitungszeiten für 2 PGF-Projekte von bis zu 2 Jahren, bevor die Förderanträge eingereicht werden konnten [22, 35]. Konkret beinhaltet der Kontaktaufbau v. a. auch eine angepasste Sprache und geeignete Formate für Informationen und Ansprache, die Entwicklung alternativer Begegnungsformen (z. B. Nachbarschaftsspaziergänge) sowie den Aufbau von Unterstützungsmöglichkeiten (z. B. Kinderbetreuung, Transportmöglichkeiten), um Freiräume und Möglichkeiten für die Teilnahme an Forschung zu schaffen. Die Bedeutung von Beziehungsaufbau und der -gestaltung wird im Community-based Participatory Research Conceptual Model (CBPR-Modell) unterstrichen. Dieses Modell stellt einen Reflexionsrahmen für partizipative Projekte dar, der Aspekte der Planungs- und Prozessqualität mit den Wirkungen auf individueller und kollektiver Ebene verknüpft. Im Zuge einer umfassenden empirischen Überprüfung des CBPR-Modells wurden Charakteristika der Beziehung (v. a. Respekt, Vertrauen, Dialog, Zuhörbereitschaft, Konfliktmanagement) als wesentliche Einflussfaktoren auf den späteren Outcome belegt [36]. In der Literatur zu partizipativer Forschung werden dagegen die Bedingungen, unter denen sich Vertrauen bildet, weniger diskutiert. Emmel et al. haben hier v. a. Empathie, Glaubwürdigkeit, ein harmonisches Verhältnis sowie die Zeit/Dauerhaftigkeit als Voraussetzungen identifiziert [26]. In diesem Zusammenhang kann die Erhebung von Daten zur Bestimmung des sozioökonomischen Status kontraproduktiv wirken.

Es werden verschiedene Zugangswege zu Forschungsteilnehmenden beschrieben, u. a. wird der Zugang über Communitys, die hier als lebensweltliche Gemeinschaften mit einem Zusammengehörigkeitsgefühl verstanden werden, als vielversprechend beschrieben [37–40]. Neben Communitys werden vor allem Zugänge über sogenannte Türöffner (Multiplikatoren) sowie die Suche nach dem Schneeballprinzip empfohlen [31, 41, 42], allerdings kann es beim Zugang über Multiplikatoren zu Selektionsmechanismen kommen [26, 43]. Darüber hinaus hat sich die Vernetzung mit relevanten Akteuren als sinnvoll erwiesen, um gemeinsam geeignete Räume für partizipative Forschung zu schaffen [44].

## Reflexion von Erfahrungen in partizipativen Forschungsprojekten

Anhand von 3 Fallstudien beleuchten wir, wie Eltern von Vorschulkindern im Berliner Bezirk Marzahn-Hellersdorf und in Lauchhammer, einer Kleinstadt in Brandenburg, ältere Menschen in Kassel sowie betreuende Angehörige von Erwachsenen mit Lernschwierigkeiten und Verhaltensauffälligkeiten im Nordosten Englands für die Partizipation an Forschung gewonnen wurden.

### Eltern fragen Eltern – ElfE

Das Forschungsprojekt ElfE arbeitete mit Eltern mit Kindern im Vorschulalter. Schuleingangsuntersuchungen in Deutschland zeigen regelmäßig Zusammenhänge zwischen dem SES der Kinder und ihrer Gesundheit [45]. Die partizipativ entwickelte Forschungsfrage lautete: Wie kann die Entwicklung von Kindern durch den Besuch von Kindergärten besser unterstützt werden (vgl. [20])? Es gab 2 Fallstudien, die in Regionen mit einem hohen Anteil von Menschen angesiedelt waren, die Transferleistungen bezogen. In Marzahn-Hellersdorf bestehen langjährige Erfahrungen mit partizipativen Initiativen und innovativen Projekten, in Lauchhammer gibt es dagegen nur wenige Angebote für Eltern und Kleinkinder – ein Unterschied, der sich für die Ansprache *selten Gehörter* als bedeutsam erweisen sollte.

Zunächst wurde in beiden Fallstudien u. a. mit der lokalen Verwaltung (z. B. dem Jugendamt) und mit lokalen Akteuren sowie Selbstvertretungen (z. B. der bezirkliche Elternausschuss Kita), die – soweit vorhanden – direkten Kontakt zu Eltern haben, eine Steuerungsgruppe aufgebaut.

Unterstützt durch die verantwortlichen Sozialarbeiterinnen fanden in Marzahn-Hellersdorf eine Fokusgruppendiskussion sowie eine gemeinschaftliche Auswertung mit jungen Eltern statt, die in ihrer Berufsausbildung unterstützt werden [46]. Daraus resultierte letztlich eine Veränderung des Forschungsfokus. Dieser hatte sich zunächst auf den Übergang vom Elternhaus in die Kita gerichtet. Die beteiligten Eltern favorisierten aber, die gesamte Kitazeit in den Blick zu nehmen. Außerdem wurde die Entwicklung eines Projektflyers zur Ansprache weiterer Eltern ebenfalls durch Eltern unterstützt.

Aufgrund der unterschiedlichen örtlichen Voraussetzungen entwickelte sich auch der weitere Weg zu den Eltern unterschiedlich:

- In Marzahn-Hellersdorf haben vor allem die kooperierenden Einrichtungen (Familienzentrum, Migrantenorganisation) ihnen bekannte Eltern persönlich zur Mitarbeit im Projekt motiviert.
- In Lauchhammer verteilten wir Flyer und Einladung über den Verteiler des regionalen Netzwerks *Gesunde Kinder* und stellten Informationen auf Facebook und Youtube ein. Ein öffentliches Briefing der lokalen Presse führte zu Beiträgen auf einem regionalen Videokanal sowie in 2 Onlinezeitungen.
- Die Kontaktaufnahme zu Kitaleitungen führte dagegen in beiden Fallstudien nicht zu einer Kooperation. Neben anderen Erklärungen (v. a. fehlende zeitliche Ressourcen, die Offenheit des Prozesses, andere Prioritätensetzung) kann dies auch daran gelegen haben, dass die Mitarbeit im Projekt als Kritik an der Kita gesehen wurde (vgl. den Beitrag Schaefer und Narimani in diesem Themenheft).

An den Informationsveranstaltungen haben in Marzahn-Hellersdorf 14 Elternteile (mehr als geplant) und in Lauchhammer 2 Elternteile (weniger als geplant) sowie 2 Elternvertretungen teilgenommen. Alle Eltern konnten auch für die weitere Zusammenarbeit gewonnen werden. In Marzahn-Hellersdorf kamen noch 5 weitere Elternteile hinzu, die von bereits involvierten Elternteilen angesprochen wurden. In Lauchhammer konnten wir über einen Flohmarkt für Kinderkleidung gemeinsam mit den bereits gewonnenen Eltern eine weitere Mutter für die kontinuierliche Mitarbeit erreichen.

Das Spektrum der beteiligten Eltern war breit: von Eltern mit russischsprachigem Migrationshintergrund und Alleinerziehenden über Eltern, die Transferleistungen und/oder Unterstützung bzw. Beratung seitens des Jugendamtes bei der Erziehung ihrer Kinder erhielten, bis hin zu Vater-Mutter-Konstellationen mit regelmäßigem Einkommen.

Für die erfolgreiche Ansprache *selten Gehörter* war aus unserer Sicht in ElfE einerseits die Anpassung des Forschungsfokus an die Interessen der Menschen aus der Lebenswelt bedeutsam. Der Vergleich zwischen beiden Fallstudien weist andererseits auf den Einfluss systemischer Barrieren hin. In Marzahn-Hellersdorf konnten mit bestehenden Einrichtungen Kooperationen aufgebaut werden, die sich dann für die Ansprache von Eltern engagiert haben (Türöffner/Multiplikatoren). Zudem entwickelten sich Schneeballeffekte, nachdem erste Eltern gewonnen waren. Diese persönliche Ansprache durch bereits bekannte (und ggf. vertraute) Fachkräfte fehlte in Lauchhammer. Trotz recht breiter Verteilung des Flyers bzw. öffentlicher Informationen zu ElfE – auch über neue Medien – konnte die Distanz zur Forschung in Lauchhammer nur mit geringem Erfolg überwunden werden.

### Gesunde Stadtteile für Ältere – Age4Health

Das Projekt Age4Health führte eine von 2 Fallstudien im Stadtteil Kassel-Bettenhausen (2015–2020) durch, um mit älteren Menschen partizipativ zu untersuchen, wie ein Quartier inklusiv entwickelt werden kann, sodass auch vulnerable ältere Menschen Zugang finden [21].

Kassel-Bettenhausen beherbergt eine vielfältige Bevölkerung mit überwiegend niedrigem und mittlerem sozioökonomischen Status. Wir konnten Age4Health im Seniorennachbarschaftszentrum Agathof ansiedeln, in dem bereits ältere Menschen aus der ganzen Stadt zu soziokulturellen Aktivitäten in meist selbst organisierten Gruppen zusammenkamen, das aber kaum als offener Nachbarschaftsraum genutzt wurde. Als Steuerungsgruppe (Leitung des Agathofs, Altenhilfereferat der Stadt Kassel, Hochschulteam) kamen wir deshalb überein, die nachbarschaftliche und inklusive Funktion des Zentrums zu stärken.

Da wir einen direkten Zugang zu *selten gehörten Älteren* anfangs nicht fanden, versuchten wir die älteren Bettenhäuser*innen über einen vielschichtigen Prozess einzubinden. Zu regelmäßigen *Runden Tischen* wurden 2‑ bis 3‑mal jährlich professionelle und zivilgesellschaftliche Akteure und die Adressatengruppe eingeladen; hier wurden lokale Ressourcen, Probleme sowie Ideen für Veränderungen identifiziert. An den Diskussionen beteiligten sich nicht alle anwesenden Älteren gleichermaßen, einige wechselten zwischen Engagement und Rückzug, manche äußerten sich selten, kamen aber regelmäßig. Die aktive Beteiligung älterer Bürger*innen nahm aber allmählich zu, als die Beteiligten sich besser kennenlernten, mit Diskussionsroutinen vertraut wurden und Selbstvertrauen gewannen, sich öffentlich zu äußern. Veränderungsideen für das Quartier wurden entwickelt, diskutiert (auch in Kleingruppen, um die Chancen für alle zu erhöhen, gehört zu werden) und nach Abstimmungen entweder fallengelassen oder umgesetzt.

Entwickelt am *Runden Tisch* konnten u. a. die beiden folgenden Initiativen zu breiterer Beteiligung beitragen:

Gemeinsam mit der Steuerungsgruppe bereiteten ältere Bürger*innen Spaziergänge in 2 Quartiersbereichen vor und legten die Routen fest. Einladungen zur Teilnahme wurden in der Nachbarschaft verteilt und privat weitergegeben. Mit einem Handkarren wurden Klappstühle und Getränke transportiert, damit auch eingeschränkte Ältere mitgehen konnten. „Problemorte“ wurden besucht und mögliche Veränderungen diskutiert. Viele ältere Menschen, vormals nicht beteiligt, nahmen an diesen Spaziergängen und später am *Runden Tisch* teil, wo die Ergebnisse diskutiert und Prioritäten abgestimmt wurden. Einige Änderungsvorschläge wurden von der Kommunalverwaltung umgesetzt (Straßenquerungen erleichtert, Bänke aufgestellt usw.), was zu einem gewissen Stolz der Beteiligten beitrug und ihre Identifikation mit dem Prozess stärkte.

Informelle Begegnungsmöglichkeiten fehlten den Älteren; so entstand als weitere Initiative die Idee eines Nachbarschaftscafés. In Räumen des Agathofs wurde ein wöchentliches, kostengünstiges Café dauerhaft eingerichtet. Ältere Freiwillige kümmerten sich um die Organisation; eine weitere Gruppe übernahm es, regelmäßig Kuchen zu backen. *Café Agathe* wird nun wöchentlich von 40 bis 50 älteren Menschen vorwiegend aus der Nachbarschaft besucht; es ist offen für Neue und knüpft an das Programm des Agathofs an; soziale Netzwerke werden gestärkt, Integration und praktische Teilnahme ermöglicht. *Café Agathe* entstand im Forschungsprozess; jedoch teilen die Café-Organisator*innen und -Besucher*innen kein explizites Forschungsinteresse mit der Steuerungsgruppe des Projekts.

In Age4Health konnten durch Schneeballprozesse und unterschiedliche Beteiligungsformate kontinuierlich mehr ältere Bürger*innen (lt. dem Leiter des Agathofs viele alte und hochaltrige Frauen, weniger Männer, mit vorwiegend einfachem Schulabschluss und unterbrochenen Berufsbiografien) in partizipative Prozesse und Aktivitäten in der Nachbarschaft einbezogen werden. Der Agathof wurde dadurch zu einem stärker quartiersorientierten, inklusiveren und teilweise selbst organisierten Ort. Die lokalen Voraussetzungen konnten über Runde Tische und Nachbarschaftsspaziergänge gestärkt werden; damit konnte lokales Wissen für Veränderungsprozesse generiert und umgesetzt werden.

### Family Based Positive Support – FaBPos

Der Alltag betreuender Angehöriger von Erwachsenen mit Lernschwierigkeiten und Verhaltensauffälligkeiten in England ist komplex; Isolation und ungedeckte Unterstützungsbedarfe sind häufig; wirksame Unterstützungsangebote fehlen. Vor diesem Hintergrund sollten mit dem FaBPos-Forschungsprojekt im Nordosten Englands Erkenntnisse für die Stärkung betreuender Angehöriger gewonnen werden. Methodische Grundlagen sollten der Achtsamkeitsansatz sowie eine Akzeptanz- und Bindungstherapie (Acceptance and Commitment Therapy [ACT]) sein.

In England werden Co-Forschende meist über Kontakte im Rahmen von Institutionen bzw. Versorgungsangeboten erreicht. Während Kinder mit Lernschwierigkeiten im Bildungssystem erfasst sind, können sie anschließend aus dem Blickfeld geraten [47]. Das Projekt suchte daher die Zusammenarbeit mit isolierten betreuenden Angehörigen.

Mit betreuenden Angehörigen, zu denen bereits Kontakt bestand, wurde zunächst ein „Core-Design-Team“ gebildet. Dieses Team entwickelte dann Gespräche bzw. Gruppendiskussionen mit weiteren betreuenden Angehörigen. Gespräche fanden in deren privaten Wohnungen (insgesamt 8 Gespräche), die Gruppendiskussionen in lokalen Familienbetreuungszentren (4 Treffen mit 12–25 Angehörigen pro Gruppe) statt. Im Zentrum stand die Frage nach der Einführung der ACT, wie isolierte betreuende Angehörige erreicht und ihr Engagement unterstützt werden könnte. ACT war einigen Angehörigen bekannt, wurde partiell aber als nebulöser „Hippieunsinn“ eingeschätzt.

Aus den Diskussionen wurde Folgendes gelernt:

- Bisherige Angebote wurden von den betreuenden Angehörigen als bürokratisch und wenig responsiv wahrgenommen und führten zu Misstrauen gegenüber Fachkräften. Eine Einladung durch Fachkräfte zur Teilnahme an der Forschung schien daher wenig ratsam.
- Vorerfahrungen mit Forschungsprojekten hatten zur Ablehnung der Teilnahme an Tests, insbesondere mit Checklisten zur psychischen Gesundheit geführt. Sie wurden als übergriffig, zeitaufwendig und nutzlos empfunden.
- Vertrauenswürdige Angebote für eine zeitliche Entlastung als Voraussetzung für eine Teilnahme fehlten. Durch die Betreuung von Angehörigen im eigenen Haushalt waren soziale Netzwerke reduziert; verbliebene Freunde und Familienmitglieder fühlten sich oft überfordert.
- Ein Interesse an Forschung richtete sich eher auf Bedürfnisse der von ihnen betreuten Angehörigen oder auf die Nutzung ihrer persönlichen Erfahrungen für andere betreuende Angehörige. Ein zeitaufwendiges Engagement zum eigenen Nutzen fanden sie schwer zu rechtfertigen. Forschung müsse an einem Ort stattfinden, an dem sie sich wohlfühlen würden.

Mit diesen Ergebnissen wurde das Projekt weiterentwickelt.

In dem im Core-Design-Team entwickelten Flyer wurde der Hinweis auf *Familien helfen Familien* und das Wissen der betreuenden Angehörigen in den Mittelpunkt gestellt. Zur praktischen Unterstützung der Angehörigen wurde außerdem ein Programmteil integriert, der Ressourcen zum Umgang mit herausfordernden Verhaltensweisen vermitteln sollte. Der Flyer wurde über soziale Medien verbreitet; eine bis dato isolierte Person wurde auf diesem Weg erreicht. Die anderen Teilnehmenden wurden über ihre Verbindung zu Einrichtungen, Fachkräften sowie vertrauten Privatkontakten erreicht. Für die Entscheidung zur Teilnahme waren das Angebot einer Betreuung ihrer Angehörigen sowie das neu implementierte Programm bedeutsam.

An FaBPos nahmen insgesamt 18 betreuende Angehörige zwischen 30 und 70 Jahren teil. Viele hatten weitere Herausforderungen zu bewältigen, z. B. die Pflege der Eltern. Nur 3 Angehörige waren berufstätig, was u. a. auf ihre hohe Alltagsbelastung hinweist. Die ursprüngliche Projektplanung wurde mithilfe der Adressat*innengruppe methodisch wie inhaltlich erheblich verändert und angepasst: v. a. wurden zunächst vorgesehene standardisierte Vorher-nachher-Tests fallen gelassen und das gesamte Projekt als diskursiver, an den Ressourcen und Interessen der Teilnehmenden ausgerichteter Prozess gestaltet.

## Diskussion

Partizipative Ansätze orientieren sich an den Lebenswelten und Bedürfnissen benachteiligter Gruppen [18]. Dennoch ist der Zugang zu *selten Gehörten* eine Herausforderung [16, 48].

Auch diese Menschen an Forschung zu beteiligen, schließt Wissenslücken und wirkt Ungleichheiten bei sozialer und gesellschaftlicher Teilhabe entgegen. Die Forschung trägt dazu bei, denjenigen Chancen zur Teilnahme zu geben, „… die am meisten zu gewinnen hätten“ [14]. Dieser Beitrag konzentriert sich auf Forschungsprojekte, die durch Wissenschaftler*innen initiiert wurden, im Gegensatz zu von Communitys selbst initiierten Prozessen. Die geschilderten 3 Fallstudien illustrieren in verschiedenen Settings und bei verschiedenen Adressatengruppen unterschiedliche Wege, wie Menschen einbezogen werden können, die bislang nicht mit Forschung erreicht werden konnten. Ein allgemeingültiges „Rezept“, wie PGF sie zuverlässig erreichen kann, ist nicht entstanden. Dennoch zeigen sich, in der Literatur und in den Fallstudien, abhängig von den sozialen und lokalen Gegebenheiten, vielversprechende Zugangswege.

Das gemeinsame Ziel der dargestellten Forschungsprojekte war die Stärkung gesundheitlicher Chancengleichheit u. a. durch die Verringerung epistemischer Ungerechtigkeit. In unterschiedlicher Weise haben die Projekte systemische Barrieren reduziert und Voraussetzungen verbessert. Unsere Erfahrungen bestätigen verschiedene Empfehlungen aus der Literatur, wie sie beispielsweise für die Einbeziehung „schwer Erreichbarer“ in gesundheitsbezogene und soziale Angebote gegeben werden (vgl. [32]). Es sind insbesondere folgende Elemente, die sich für das Erreichen *selten Gehörter* als bedeutsam erwiesen haben:

Der Aufbau vertrauensvoller Beziehungen überbrückt die Distanz und ggf. das Misstrauen gegenüber der Wissenschaft. Auf der Basis tragfähiger Kooperationen können zudem die Ressourcen lokaler Kooperationspartner*innen, die eher als die Wissenschaft über informelle Systeme und Routinen verfügen, genutzt werden, um Zugangswege zu entwickeln. Auch verfügen entsprechende Personen oft über vertrauensvolle Kontakte. Insofern sollten Kooperationen möglichst mit unterschiedlichen Akteuren aufgebaut werden.

Ausgehend von einzelnen Menschen aus der Lebenswelt können über Schneeballprozesse weitere für eine Beteiligung gewonnen werden. Eine lokale Infrastruktur mit Einrichtungen, die in Kontakt zu der Adressatengruppe stehen, und mit Interessensvertretungen, wie z. B. Seniorenbeiräten, ist deshalb eine wichtige Ressource. Fehlen solche Strukturen, sollte die Initiierung eines partizipativen Projektes überdacht werden.

Partizipation ist als inkrementeller Prozess zu verstehen, als „alltäglicher“ und nicht einmaliger Vorgang, der die Forschung grundlegend prägt. Dieser Prozess braucht Zeit, nicht nur, um Wege zu finden und die Distanz zur Forschung zu überwinden. Auch kann das Konzept der Partizipation für Menschen in ihrer Lebenswelt fremd sein, sodass eine Vorstellung dazu erst entwickelt werden muss. Es braucht Selbstvertrauen, den eigenen Sichtweisen und Erfahrungen Bedeutung beizumessen. Nach Holcombe besteht zwischen Partizipation und Empowerment eine „untrennbare“ Verbindung [49].

Nicht zuletzt bedarf es der Bereitschaft der Wissenschaft, Kontrolle abzugeben (z. B. über die Festlegung des Forschungsthemas) und auf das Wissen und die Erfahrungen anderer zu vertrauen. Dies kann nicht nur ungewohnt sein, sondern auch zu verschiedenen Dilemmata führen. Nicht nur für den Förderantrag ist der Fokus der Forschung festzulegen. Auch für die erste Ansprache der Menschen aus der Lebenswelt und möglicher Kooperationspartner ist es wichtig, ein Thema zu benennen. Wie in 2 der Fallstudien gezeigt, trifft das vordefinierte Thema oder der methodische Ansatz möglicherweise nicht auf die Interessen der Menschen in der Lebenswelt. Auch kann eine Veränderung von Forschungsfokus und geplanten Methoden zu Konflikten mit Kooperationspartnern oder Fördermittelgebern führen. Deshalb sollten vorab z. B. über Gespräche und/oder Fokusgruppendiskussionen mit der Adressat*innengruppe die Forschungsinteressen eingegrenzt und erst danach weiter kommuniziert werden. Partizipative Forschung mit *selten Gehörten* bedarf daher einer vielseitigen Vorbereitung, bevor die eigentliche Forschung beginnen kann. Wir schließen damit an Greenhalgh et al. an, nach denen die „am schwersten zu erreichenden Gruppen möglicherweise unerreicht bleiben“, wenn die Ressourcen fehlen oder Forschende nicht in der Lage oder bereit sind, in entsprechende Beziehungen zu investieren [50]. In dieser Hinsicht war die Förderung des Forschungsverbundes PartKommPlus beispielhaft, da dieser insgesamt für 6 Jahre gefördert wurde.

Abschließend möchten wir auf zweierlei Limitationen hinweisen. Wir konnten nur ansatzweise bestimmen, welche Menschen beteiligt waren, weil wir die Indikatoren des sozioökonomischen Status (SES) von den Beteiligten nicht erhoben haben. Dies hätte stigmatisierend wirken und die Zusammenarbeit *auf Augenhöhe *konterkarieren können. Allerdings ist der SES nur *ein* Indikator für eine Marginalisierung. Es sollte daher weiter nach Lösungen gesucht werden, wie sich individuelle Belastungen und Herausforderungen in möglichst wenig stigmatisierender Weise beschreiben lassen.

Die Bedeutung von Türöffnern/Multiplikatoren hat sich in unseren Ergebnissen bestätigt; Selektionseffekte können aber nicht ausgeschlossen werden bzw. wurden nicht überprüft. Dies wäre ein weiterer Anknüpfungspunkt für künftige Forschung.

## Fazit

*Selten Gehörte* einzubeziehen, bleibt eine Herausforderung – nicht nur in der partizipativen Forschung. Zugleich wird die Frage, wie dies gelingen kann, bislang in akademischen Publikationen, auch innerhalb der partizipativen Forschung, selten thematisiert. Wir wünschen uns weitergehende Diskussionen zu dieser Herausforderung, gerade auch um Wege zu finden, gesundheitliche Ungleichheiten zu verringern.

Wir konnten zeigen, dass die partizipative Gesundheitsforschung in der Lage ist, *selten Gehörte* einzubeziehen. Mit kleinen Schritten und durch den Aufbau vertrauensvoller Beziehungen können Barrieren abgebaut und Grenzen schrittweise erweitert werden. Allerdings erfordert dies Zeit, vielfältige Interaktionen, zuverlässige Zusammenarbeit, langfristige Präsenz vor Ort, eine Abgabe von Kontrolle und sorgfältige Reflexion der Formen des gemeinsamen Handelns.

Die abgeleiteten Schlussfolgerungen könnten als Zumutung gesehen werden:

- für Wissenschaftler*innen, den Forschungsfokus und -prozess nicht immer unter Kontrolle zu haben, sich auf einen offenen Prozess einzulassen, von dem zunächst nicht absehbar ist, in welche Richtung er sich entwickelt;
- für Kooperationspartner, die zunächst nicht wissen können, ob ihre Anliegen Berücksichtigung finden;
- für Fördermittelgeber, die in langwierige und komplexe Prozesse investieren, ohne vorab klar messbare Ergebnisse absehen zu können.

Wir sehen jedoch in der partizipativen Forschung ein gutes Potenzial, gesundheitliche Chancengleichheit zu stärken und wissenschaftlich fundiert voranzubringen, auch indem bislang bestehende Wissenslücken gefüllt werden.
